# Epidermal growth factor receptor inhibitor therapy for recurrent respiratory papillomatosis

**DOI:** 10.12688/f1000research.2-202.v1

**Published:** 2013-10-03

**Authors:** Matthew M. Moldan, Bruce C Bostrom, Robert J Tibesar, Timothy A. Lander, James D. Sidman

**Affiliations:** 1 Children’s Hospitals and Clinics of Minnesota, Minneapolis, MN 55404, USA; 2 Department of Otolaryngology, University of Minnesota, Minneapolis, MN 55455, USA

## Abstract

The epidermal growth factor pathway has been implicated in various tumors, including human papillomavirus (HPV) lesions such as recurrent respiratory papillomatosis (RRP). Due to the presence of epidermal growth factor receptors in RRP, epidermal growth factor receptor (EGFR) inhibitors have been utilized as adjuvant therapy. This case series examines the response to EGFR inhibitors in RRP. Four patients with life-threatening RRP were treated with EGFR inhibitors. Operative frequency and anatomical Derkay scores were calculated prior to, and following EGFR inhibitor treatment via retrospective chart review. The anatomical Derkay score decreased for all four patients after initiation of EGFR inhibitor therapy. In one patient, the operative frequency increased after switching to an intravenous inhibitor after loss of control with an oral inhibitor. In the other patients there was a greater than 20% decrease in operative frequency in one and a more than doubling in the time between procedures in two.  This study suggests that EGFR inhibitors are a potential adjuvant therapy in RRP and deserve further study in a larger number of patients.

## Introduction

Recurrent respiratory papillomatosis (RRP) occurs with human papillomavirus (HPV) infection of the respiratory tract epithelium, typically by HPV types 6 and 11 ^1, 2^. It is the most common benign neoplastic laryngeal disorder in children ^3^. RRP has been associated with an increased risk of airway obstruction ^4^. Juvenile onset RRP is more severe and results in more intensive therapy ^2^. The mainstay of RRP management remains surgical debulking; however, adjuvant therapies are offered in up to a fifth of cases of RRP ^5^.

As a result of the low prevalence of RRP, large controlled trials of adjuvant therapies have been limited. Epidermal growth factor receptor (EGFR) inhibitors have been used as an adjuvant therapy due to the presence of EGFR in papillomas ^6^. This case series examines the use of EGFR inhibitors in four patients with life-threatening RRP.

## Materials and methods

This was an institutional review board (IRB) approved study to examine the response to EGFR inhibitors in RRP. A waiver of informed consent and an IRB waiver of HIPPA authorization were approved by the IRB prior to the advent of the study as the data was anonymised. Those patients with severe RRP, defined as requiring more than four surgical procedures per year with rapid regrowth of papilloma leading to airway compromise, who had been treated with EGFR inhibitors, were identified and their medical charts reviewed. The interval between operations was based upon the physicians’ intraoperative determination of disease burden and was not determined by a specific protocol. Surgical procedures used included carbon dioxide (CO _2_) laser and microdebrider or a combination thereof. The operative notes were reviewed to determine a modified Derkay Severity Score, utilizing the anatomical portion of the scoring system, at the time of each surgical debulking ^7^. The EGFR inhibitors used, all of which are FDA approved for EGFR expressing malignancies, included erlotinib (Tarceva®), gefitinib (Iressa®), and panitumumab (Vectibix®). Erlotinib (starting dose of 85 mg/m ^2^ PO rounded to nearest 12.5 mg) or gefitinib (starting dose of 325 mg/m ^2^ PO rounded to nearest 50 mg) were administered daily while panitumumab (starting dose of 150 mg/m2 IV) was given immediately following each operation with a minimum of every two weeks. An oral medication was initiated first (gefitinib if available or erlotinib if gefitinib was not available). If there was a concern for oral bioavailability or inadequate response to the oral EGFR inhibitor, patients were then transitioned to IV panitumumab. Informed consent was obtained for all patients.

## Results

Four patients from 2003 through 2012 met the criteria listed above. Table 1 includes patient demographic and disease-specific information. EGFR expression and associated grade was determined by immunohistochemical analysis prior to initiation of EGFR inhibitor therapy and is also reported in Table 1 ^8^. The outcome measures for each patient following adjuvant therapy were compared to their own measures prior to therapy; therefore the diversity in regard to age, viral type, and number of operations within the patient group had no effect on outcome.

**Table 1.** Patient demographics, virus characteristics, pathology results, and treatment modalities.

Patient No./sex/age	HPV type	EGFR grade ^9^	Current adjuvant treatment	Duration of current treatment (months)
**1/F/3**	6	3	Panitumumab	15 on & 12 off
**2/M/6**	6 & 84	2–3	Panitumumab	23
**3/F/7**	11	2–3	Panitumumab	20
**4/M/23**	11	3	Gefitinib	118 on & 2 off

M=Male, F=Female, EGFR=Epidermal Growth Factor Receptor.

The outcome data is included in Table 2. Prior to start of adjuvant therapy the Derkay scores of three of the patients were increasing. Following initiation of the EGFR inhibitor therapy, the Derkay score decreased for all four patients. In one patient, the operative frequency actually increased. She was previously well controlled on oral gefitinib but her condition acutely worsened. This prompted a switch to intravenous panitumumab. Contrastingly, in another patient there was a greater than 20% improvement (decrease) in the operative frequency. In the remaining two patients the time interval between procedures more than doubled.

**Table 2.** Treatment and outcome data.

			Operation frequency (days between procedures)*		Anatomic Derkay score*	
Patient	Previous treatments	Current treatment	Before treatment	After treatment	Before treatment	After treatment
1	Erlotinib/celecoxib (oral)	Panitumumab (intravenous)	12	26	18	6
2	Erlotinib/celecoxib (oral)	Panitumumab (intravenous)	14	17	15	10
3	Erlotinib (oral), Interferon alfa-2a (subcutaneous), Gefitinib (oral)	Panitumumab (intravenous)	28	16	6	4
4	Interferon alfa-2a (subcutaneous), tretinoin (oral), ribavirin (oral and inhaled), indole-3-carbinol (oral)	Gefitinib (oral)	7	25	9	5

* Data are reported as median.

*Patient 1*. A 3 year old female was diagnosed at 6 months of age and was solely managed surgically until 14 months of age. Prior to panitumumab treatment her Derkay score was increasing and she required weekly operations. She had previously received a trial of erlotinib and celecoxib with minimal effect. She had 23 operations prior to starting IV panitumumab at a dose of 200 mg/m ^2^.

Following the switch, her operation frequency decreased, with an operation every 26 days and modified Derkay score of 6 on average ( Table 2). The patient has undergone 15 IV treatments with some minimal side effects. These have included a skin rash that decreased with ongoing therapy and resolved off therapy, diarrhea limited by probiotic use, an MRSA leg abscess and untreated iron deficiency without anemia that resolved off therapy. During the most recent operation, her airway was free from papillomas and panitumumab treatment was discontinued and the patient has remained free of recurrence off therapy for 12 months.

*Patient 2*. A 6 year old male was diagnosed with RRP at the age of 2. He received nine microdebrider procedures at an outside hospital prior to referral to us for adjuvant therapy. He was first started on erlotinib and celecoxib, which failed to control his disease. The patient had received 30 surgical procedures prior to initiation of IV panitumumab at a dose of 130 mg/m ^2^, later increased to 200 mg/m ^2^ to improve response.

Following panitumumab treatment, his operative frequency and Derkay score have decreased ( Table 2). Side effects have included increased thirst, nocturnal enuresis, an acneform rash and untreated iron deficiency without anemia. His height has fallen from the 50 ^th^ to 25 ^th^ percentile over two years while on panitumumab. The patient began the HPV vaccination series a few months after starting panitumumab. He has also received diindolylmethane (DIM, Bioresponse®) 150 mg 2–3 times daily. Due to a recent improvement in his condition, his surgical interval has been lengthened to approximately twelve weeks.

*Patient 3*. A 7 year old female was diagnosed at 9 months. Shortly thereafter she received a tracheostomy and G-tube at an outside institution due to the extent of the RRP. Her condition continued to worsen and her care was transferred to our institution for adjuvant therapy.

After failure of a short trial of erlotinib (60 mg/m ^2^ daily for 1 month followed by 85 mg/m ^2^ daily for 1 month) she was managed briefly with interferon alpha 1.5 million units by subcutaneous injection daily prior to initiation of gefitinib at an initial dose of 325 mg. She was managed with gefitinib and surgical debulking for nearly five years. The patient was discovered to be non-compliant once in the past with worsening of her modified Derkay score after which her mother closely monitored gefitinib administration. More recently, her RRP acutely worsened after which it was decided to switch to IV panitumumab at a dose of approximately 150 mg/m ^2^ (later increased to 200 mg/m ^2^). The patient had received 69 laser procedures and four requiring combined mechanical and laser debridement prior to panitumumab.

Side effects have included a rash, increased urinary frequency and nocturnal enuresis, and occasional mild fevers with no known source. Her supraglottic disease was so severe that she was only being treated, and thus scored, for tracheal disease. She was also noted to have progressive iron deficiency without anemia that did not respond to oral iron, though it did improve with IV iron sucrose. She has been less than 5 ^th^ percentile for height but continues to grow. A recent bone age study showed delay. After another period of worsening RRP, panitumumab was increased to 265 mg/m ^2^ and DIM was added. Subsequently oral propranolol was added at 3 mg/kg/day divided into three doses per day (TID) without effect after more than a month. Subsequent to this regime, the panitumumab dose was decreased to 200 mg/m ^2^ and daily gefitinib (270 mg/m ^2^/day) restarted with no significant change in operative frequency.

*Patient 4*. A 23 year old male was diagnosed at the age of 3 months. His case has been reported in a previous study ^6^. The patient’s comorbidities include interferon-induced nephrotic syndrome with stage III acute renal failure from focal segmental glomerulosclerosis, hypertension and developmental delay. He was born at 28 weeks gestation. He required a tracheostomy as a result of extensive laryngeal stenosis secondary to RRP. The patient was managed with interferon alfa-2a until approximately 8 years of age.

Due to life-threatening airway obstruction 10 years ago, he was started on gefitinib at an initial dose of approximately 400 mg/m ^2^/day divided twice daily because it was thought the patient would not survive. His Derkay score was based upon the trachea and bronchi only as these were the areas being treated due to complete stenosis of the larynx.

Adverse effects have included acne-form rash, dry skin, and minimal diarrhea. He also developed iron deficiency anemia two years ago requiring iron infusions and occasional blood transfusion. An extensive workup has not found a source of blood loss. He continued to receive gefitinib with satisfactory control of his RRP until June 2013 when a trial period off gefitinib was started in conjunction with increased surgical debridement at the family’s request.

## Discussion

Our interest in EGFR inhibitors stemmed from the benefit observed with the use of gefitinib in a patient with life-threatening RRP (patient 4 above) ^6^. Three EGFR inhibitors—panitumumab, gefitinib, and erlotinib—have been utilized in these four patients. In our hands, decrease in papilloma severity has been observed only with panitumumab and gefitinib. The lack of response to erlotinib is surprising given that it targets EGFR and there is a prior report of response to erlotinib and celecoxib ^9^. Interestingly there is one prior report of the use of intravenous cetuximab, another EGFR inhibitor, in RRP without effect ^10^. The use of EGFR inhibitors in the treatment of RRP is off-label. However, due to the EGFR expression in these patients’ papillomas it was hypothesized that EGFR antagonists could offer benefit.

This case series is justifiably limited in scope; it nevertheless suggests that EGFR inhibitors are a potential adjuvant therapy for the treatment of RRP. Beneficial results were observed in all four patients as evidenced by improvement in the Derkay score. In three of the four patients the operative frequency decreased, with the time interval between operations lengthening to twice what was required prior to EGFR inhibitor therapy in two subjects.

None of the patients experienced serious adverse effects from the medications with minimal tolerable adverse events noted. Common side effects of EGFR inhibitors include skin rash, hypomagnesemia, paronychia, fatigue, abdominal pain, nausea and diarrhea. Two patients (patients 2 and 3) developed nocturnal enuresis on panitumumab one of which (patient 3) did not have this side effect while on gefitinib. This has not been previously described with panitumumab or other EGFR inhibitors. All patients developed iron deficiency and one severe anemia which has also not been described previously for the use of EGFR inhibitors. A possible mechanism is EGFR inhibition leading to an increase in hepcidin preventing absorption of oral iron ^11^.

Various factors could play a role in the response to adjuvant therapy. These may include: the extent of surgical excision, the method used for surgical debulking, the natural course of the disease and the aggressiveness of the virus. None of the patients have a systemic immune deficiency as an explanation for this variability. For these reasons the study of adjuvant therapies in RRP is problematic. Additionally, as with other adjuvant treatments studied for RRP, the number of patients included in this series is very limited. Larger trials comparing the efficacy of EGFR inhibitors with other promising therapies, such as intralesional cidofovir or vorinostat, are needed in order to determine the relative efficacy of these treatments ^12, 13^. EGFR inhibitors appear to improve papilloma disease control as an adjunct to surgical therapy—at least in some patients. They do not appear to eradicate papillomas and at the current time the optimal duration of therapy is unknown. The major side effect to date has been skin rash, which has been successfully managed with emollients, sunscreen for sun exposure and selective use of antibiotics for acneform lesions. Although there may be unknown risks from long-term therapy with EGFR inhibitors, our opinion is that the benefits described outweigh the possibility of future unknown risks.

## Conclusion

This case series suggests that EGFR inhibitors are a potential adjuvant therapy for the treatment of RRP as evidenced by the improvement in the modified Derkay scores and the general decrease in operative frequency.

## Consent

A waiver of informed consent and an IRB waiver of HIPPA authorization were approved by the IRB prior to advent of the study.
