# Supplementary material for: Epidermal growth factor receptor inhibitor therapy for recurrent respiratory papillomatosis
Source: F1000Res. 2013 Oct 3;2:202. [Version 1] doi: 10.12688/f1000research.2-202.v1 (PMC3999927; doi:10.12688/f1000research.2-202.v1)
Supplement: Peer review history

# Peer review: Referee response for version 1

**Published:** 2014-03-31

**Authors:** Lluís Nisa

**Affiliations:** 1 Department of Otorhinlaryngology, Hôpital de Sion, Sion, Switzerland

Juvenile-onset respiratory papillomatosis is often a severe disease, with an extraordinary burden on the quality of life of children and their families. Surgical approaches, especially debulking and, when not avoidable, tracheotomy, remain the mainstay of therapy for this devastating disease. Therefore, the need for adjuvant medical therapies that may allow long-term remission is evident ^1^^-^^3^. In this sense, the current case series by Moldan and collaborators is worthy of attention. The series also stresses the fact that several medical adjuvant treatments are available but, unfortunately, none has shown conclusive evidence in terms of efficacy.

 

The authors treated four patients with an off-label indication for EGFR inhibitors, often combining several molecules (antibodies and tyrosine kinase inhibitors) in the same patients. Furthermore, most patients had in the past received other forms of medical therapy (e.g. celecoxib, ribavirin, INF alpha-2A). It is interesting that patients initially treated with erlotininb (patients 1, 2, and 3), when switched to panitumumab responded better in two cases. This is worthy of attention, as the same holds true for the improved results of cetuximab versus erlotinib in the treatment for head and neck cancer ^4^.

 

As the authors and Dr. Bauman state, the major difficulty in the choice of a given adjuvant treatment, including EGFR inhibitors, is the lack of readouts for response stratification. As for oropharyngeal cancer, immunohistochemical evidence of EGFR overexpression is not an accurate predictor of treatment responses. The same holds true for every medical adjuvant therapy.

 

The relatively low incidence of severe RRP makes it difficult to set up proper randomized clinical trials, a problem which may be palliated by multicenter efforts.

As the authors correctly state, their findings must be considered as preliminary data which need further in-depth evaluation before any specific recommendations may be formulated.

  

As an outlook, it is likely that further understanding of the molecular pathogenesis of HPV-related tumorigenesis is going to lead to the development of new approaches, for example PI3K inhibitors (given the association between HPV and PI3K mutations in cancer) ^5^.

I have read this submission. I believe that I have an appropriate level of expertise to confirm that it is of an acceptable scientific standard.

## References

[1] BishaiDKashimaHShahK: The cost of juvenile-onset recurrent respiratory papillomatosis.*Arch Otolaryngol Head Neck Surg*.2000;126(8): 202935-93910.1001/archotol.126.8.93510922223

[2] SilverbergMJ:ThorsenPLindebergHAhdieh-GrantLShahKV: Clinical course of recurrent respiratory papillomatosis in Danish children.*Arch Otolaryngol Head Neck Surg.*.2004;130(6): 202711-71610.1001/archotol.130.6.71115210551

[3] DerkayCS: Task force on recurrent respiratory papillomas. A preliminary report.*Arch Otolaryngol Head Neck Surg.*.1995;121(12): 2021386-139110.1001/archotol.1995.018901200440087488368

[4] HansenARSiuLL: Epidermal growth factor receptor targeting in head and neck cancer : have we been just skimming the surface?. *J Clin Oncol.*.2013;31(11): 2021381-138310.1200/JCO.2012.47.922023460713

[5] NicholsACPalmaDAChowWTanSRajakumarCRizzoGFungKKwanKWehrliBWinquistEKoropatnickJMymrykSYooJBarrettJW: High frequency of activating PIK3CA mutations in human papillomavirus-positive oropharyngeal cancer.*JAMA Otolaryngol Head Neck Surg.*.2013;139(6): 202617-62210.1001/jamaoto.2013.321023787421

# Peer review: Referee response for version 1

**Published:** 2013-12-03

**Authors:** Julie Bauman

**Affiliations:** 1 Division of Hematology/Oncology, University of Pittsburgh, Pittsburgh, PA, USA

M. Moldan and colleagues report an intriguing, retrospective case series of four patients with severe recurrent respiratory papillomatosis (RRP) treated with epidermal growth factor receptor (EGFR) inhibitors. Two descriptive features, including a numerical increase in the intersurgical interval for three of four cases, and a numerical decrease in anatomic Derkay score in all four, prompted the authors’ conclusion that EGFR inhibitors have a potential adjuvant role in the management of RRP. Inclusion in this report required both severe disease (defined by the authors as requiring at least four debridements per year) and treatment with an EGFR inhibitor. Authors describe neither a systematic process for case identification, nor how many cases were excluded – raising the question of how selective reporting was avoided. 

Particular caution is warranted in clinical interpretation of the results, due to the variable natural history of RRP. The average pediatric patient requires approximately 20 lifetime surgical procedures, including airway debridement. However, 19% of patients demonstrate a more aggressive course requiring more than 40 lifetime procedures, and a small percentage of patients will spontaneously remit ^1^^-^^3^. Patterns of surgical frequency for individuals with RRP indicate significant fluctuation in intersurgical intervals, independent of adjuvant use ^4^. Moreover, the anatomic Derkay score improved in both the cidofovir and placebo groups in a randomized, controlled trial in juvenile RRP ^5^. Thus, descriptive improvements in intersurgical interval or Derkay score as noted in this selective, retrospective report must be treated with caution. This case series, along with other anecdotal reports in the literature ^6^^-^^8^, may justify a prospective, randomized trial but do not establish EGFR inhibitor therapy as an adjuvant for RRP.

The use of EGFR inhibitor therapy in RRP, including this report, has been justified by the presence of EGFR over-expression in laryngeal papillomas relative to normal laryngeal epithelium ^9^. Of note, EGFR expression by immunohistochemistry has been a disappointing biomarker of response to EGFR inhibitor therapy in cancers of the lung, colon and head and neck – areas where EGFR inhibitors are currently FDA approved ^10^^-^^12^. EGFR expression is unlikely to be an appropriate selection factor for patients with RRP to be treated with EGFR inhibitor therapy. Of particular relevance to this question, in head and neck cancer, EGFR expression is lower in cancers driven by human papillomavirus (HPV) than those driven by tobacco and other environmental carcinogens ^13^^,^^14^. Nonetheless, HPV appears to deregulate EGFR independent of expression level: in preclinical models, the E6 HPV oncoprotein increases phosphorylation of EGFR, downstream signaling, and internalization of activated receptor forms ^15^. The importance of EGFR inhibitor therapy, if confirmed in prospective, randomized trials in RRP or other HPV-related neoplasms, will likely relate to HPV biology rather than EGFR expression level *per se*. 

 

I have read this submission. I believe that I have an appropriate level of expertise to confirm that it is of an acceptable scientific standard.

## References

[1] VenkatesanNNPineHSUnderbrinkMP: Recurrent respiratory papillomatosis. *Otolaryngol Clin North Am*.2012;45(3) : 202671-69410.1016/j.otc.2012.03.00622588043PMC3682415

[2] ArmstrongLRDerkayCSReevesWC: Initial results from the national registry for juvenile-onset recurrent respiratory papillomatosis. RRP Task Force. *Arch Otolaryngol Head Neck Surg*.1999;125(7) : 202743-74810.1001/archotol.125.7.74310406310

[3] LarsonDADerkayCS: Epidemiology of recurrent respiratory papillomatosis. *APMIS*.2010;118(6-7) : 202450-45410.1111/j.1600-0463.2010.02619.x20553527

[4] OngkasuwanJFriedmanEM: Juvenile recurrent respiratory papilloma: variable intersurgical intervals. *Laryngoscope*.2012;122(12) : 2022844-284910.1002/lary.2353422847877

[5] McMurrayJSConnorNFordCN: Cidofovir efficacy in recurrent respiratory papillomatosis: a randomized, double-blind, placebo-controlled study. *Ann Otol Rhinol Laryngol*.2008;117(7) :477-4831870042110.1177/000348940811700702

[6] LimsukonASusantoIHooGWDubinettSMBatraRK: Regression of recurrent respiratory papillomatosis with celecoxib and erlotinib combination therapy. *Chest*.2009;136(3) : 202924-92610.1378/chest.08-263919736197

[7] LoyoMPaiSINettoGJTunkelDE: Agressive recurrent respiratory papillomatosis in a neonate. *Int J Pediatr Otorhinolaryngol*.2008;72(6) : 202917-92010.1016/j.ijporl.2008.02.02218423627

[8] HaoZDillardTBiddingerPPatelV: Suppression of respiratory papillomatosis with malignant transformation by erlotinib in a kidney transplant recipient. *BMJ Case Rep*.2013; 20210.1136/bcr-2013-00875723986124PMC3761685

[9] JohnstonDHallHDiLorenzoTPSteinbergBM: Elevation of the epidermal growth factor receptor and dependent signaling in human papillomavirus-infected laryngeal papillomas. *Cancer Res*.1999;59(4) :968-97410029092

[10] LicitraLMesiaRRiveraFRemenárEHittRErfánJRotteySKaweckiAZabolotnyyDBenassoMStörkelSSengerSStrohCVermorkenJB: Evaluation of EGFR gene copy number as a predictive biomarker for the efficacy of cetuximab in combination with chemotherapy in the first-line treatment of recurrent and/or metastatic squamous cell carcinoma of the head and neck: EXTREME study. *Ann Oncol*.2011;22(5) : 2021078-108710.1093/annonc/mdq58821048039PMC3082162

[11] LicitraLStörkelSKerrKMVan CutsemEPirkerRHirschFRVermorkenJBvon HeydebreckAEsserRCelikICiardielloF: Predictive value of epidermal growth factor receptor expression for first-line chemotherapy plus cetuximab in patients with head and neck and colorectal cancer: analysis of data from the EXTREME and CRYSTAL studies. *Eur J Cancer*.2013;49(6) : 2021161-116810.1016/j.ejca.2012.11.01823265711

[12] ShephardFARodrigues PereiraJCiuleanuTTanEHHirschVThongprasertSCamposDMaoleekoonpirojSSmylieMMartinsRvan KootenMDediuMFindlayBTuDJohnstonDBezjakAClarkGSantabárbaraPSeymourLNational Cancer Institute of Canada Clinical Trials Group: Erlotinib in previously treated non-small-cell lung cancer. *N Engl J Med*.2005;353(2) : 202123-13210.1056/NEJMoa05075316014882

[13] LassenPOvergaardJEriksenJG: Expression of EGFR and HPV-associated p16 in oropharyngeal carcinoma: Correlation and influence on prognosis after radiotherapy in the randomized DAHANCA 5 and 7 trials. *Radiother Oncol*.2013;108(3) : 202489-49410.1016/j.radonc.2013.08.03624060179

[14] TroyJDWeissfeldJLYoukAOThomasSWangLGrandisJR: Expression of EGFR, VEGF, and NOTCH1 Suggest Differences in Tumor Angiogenesis in HPV-Positive and HPV-Negative Head and Neck Squamous Cell Carcinoma. *Head Neck Pathol*.2013;7(4) : 202344-35510.1007/s12105-013-0447-y23645351PMC3824798

[15] SpangleJMMungerK: The HPV16 E6 oncoprotein causes prolonged receptor protein tyrosine kinase signaling and enchances internalization of phosphorylated receptor species. *PLoS Pathog*.2013;9(3) : 20210.1371/journal.ppat.100323723516367PMC3597533
